# Inertial measurement unit technology for gait detection: a comprehensive evaluation of gait traits in two Italian horse breeds

**DOI:** 10.3389/fvets.2024.1459553

**Published:** 2024-10-16

**Authors:** Vittoria Asti, Michela Ablondi, Arnaud Molle, Andrea Zanotti, Matteo Vasini, Alberto Sabbioni

**Affiliations:** ^1^Department of Veterinary Sciences, University of Parma, Parma, Italy; ^2^Italian Breeding Association for Equine and Donkey Breeds (ANAREAI), Roma, Italy

**Keywords:** equids, Equisense, IMU, genetics, machine learning

## Abstract

**Introduction:**

The shift of the horse breeding sector from agricultural to leisure and sports purposes led to a decrease in local breeds’ population size due to the loss of their original breeding purposes. Most of the Italian breeds must adapt to modern market demands, and gait traits are suitable phenotypes to help this process. Inertial measurement unit (IMU) technology can be used to objectively assess them. This work aims to investigate on IMU recorded data (i) the influence of environmental factors and biometric measurements, (ii) their repeatability, (iii) the correlation with judge evaluations, and (iv) their predictive value.

**Material and methods:**

The Equisense Motion S^®^ was used to collect phenotypes on 135 horses, Bardigiano (101) and Murgese (34) and the data analysis was conducted using R (v.4.1.2). Analysis of variance (ANOVA) was employed to assess the effects of biometric measurements and environmental and animal factors on the traits.

**Results and discussion:**

Variations in several traits depending on the breed were identified, highlighting different abilities among Bardigiano and Murgese horses. Repeatability of horse performance was assessed on a subset of horses, with regularity and elevation at walk being the traits with the highest repeatability (0.63 and 0.72). The positive correlation between judge evaluations and sensor data indicates judges’ ability to evaluate overall gait quality. Three different algorithms were employed to predict the judges score from the IMU measurements: Support Vector Machine (SVM), Gradient Boosting Machine (GBM), and K-Nearest Neighbors (KNN). A high variability was observed in the accuracy of the SVM model, ranging from 55 to 100% while the other two models showed higher consistency, with accuracy ranging from 74 to 100% for the GBM and from 64 to 88% for the KNN. Overall, the GBM model exhibits the highest accuracy and the lowest error. In conclusion, integrating IMU technology into horse performance evaluation offers valuable insights, with implications for breeding and training.

## 1 Introduction

In the last decades, there has been a significant loss in biodiversity, although is key for maintaining a sustainable environment. Biodiversity enhances animals’ resistance and resilience to stress including those caused by climate change. Biodiversity at genetic, species and ecosystem levels helps the challenges posed by distinct and changing environmental conditions and socio-economic factors. According to the Food and Agriculture Organization of the United Nations (FAO), the current rate of biodiversity loss is unprecedented in the past century, with 26% of local breeds at risk of extinction and 67% with unknown risk status (1). This is true in the equine sector as well, where most of the local breeds are considered endangered or at risk of extinction. Italian horse heritage comprises breeds reared and adapted to different regional climates, cultures, and traditions. Over the last century, the horse breeding sector has experienced significant transformations. Indeed, horses that once were bred for meat production, warfare, or agricultural purposes, are now bred for sport or leisure activities (2). Thus, Italian local breeds, historically used for meat or draft work, are facing a decrease in population size, due to the change in the market demand and a consequent loss of their original breeding purpose. For this reason, most of them are now considered endangered by FAO, which in its report declared that, in 2022, horses are among the species with the largest proportion of breeds at risk of extinction (3). Currently, Italy counts 22 distinct breeds (4), 17 of which are classified as endangered. Horses belonging to these breeds are managed by four associations: ANACAITPR (National Association of Breeders of the Italian Heavy Draft Horse), ANAMF (National Association of Breeders of the Murgese Horse and the Martina Franca Donkey), ANACRHAI (National Association of Breeders of Haflinger Horses in Italy) and ANAREAI (National Breeding Association for Equine and Asinine Breeds in Italy), which aim to maintain genetic diversity and monitor population size. However, the transition from agricultural to sport horses presents a challenge for breed conservation since it must face the need to modernize the breeds while preserving genetic diversity. As an example, in the Bardigiano horse breed, which is part of the Italian equine heritage and it is facing the need to modernize towards current market demand, genetic diversity preservation is also pivotal (5). Indeed, it has been shown that both at pedigree and genomic level inbreeding has increased in the latest generations. Therefore, breeding strategies for optimizing the contribution of breeding animals are key to ensure long-term survival of breed (6).

In a few Italian breeds, tools are available to evaluate horses based on traditional and linear scores for conformation, which are used to estimate breeding values (7). These evaluations can be used as indirect measures of movement-related traits, which might help the transition from work horses to leisure ones. However, since the modern market seeks especially sport and leisure horses (8), a comprehensive evaluation of gaits might be necessary. Currently, none of the Italian equine breeds have developed protocols to evaluate gaits and challenges exist due to the extensive training required for judges and the subjectivity involved in gait assessment. Animal breeding relies on precise phenotyping to be effective and often the phenotype recording is a limiting factor (9). Gaits traits in horses fit in this scenario of difficult traits, as they are influenced by both genetics (10) and environmental factors. In addition, these traits are exposed to change over time, and to human error and subjectivity during data collection. Consequently, the gap between objective recording and the difficulty in defining them must be filled to allow faster improvement in the breeding scheme. Therefore, there is a need to propose novel gaits’ traits that can provide objective measurements to meet the current challenges that local breeds are facing in shifting their breeding goals (11).

The integration of new tools such as inertial measurement units (IMU) technology and machine learning algorithms for image analysis offers solutions to this challenge. The IMUs are devices that measure acceleration and angular velocity, providing detailed movement data. Machine learning algorithms can analyze this data to predict and evaluate traits with greater accuracy than traditional methods. These tools are often combined to collect data efficiently and cost-effectively, requiring less time and expertise from the judges (12). Indeed, machine learning models can use data from sensor devices (13). The gold standard for kinematic analysis is the optical motion capture (OMC), which uses multiple cameras and reflective markers to track the movement of horses with high accuracy. Although this technology provides highly reliable and detailed data (14) it requires complex setups and is usually limited to research environments due to its high cost and logistical demands.

While sensors are widely used in the animal production sector for health-related monitoring (15), their application in equine performance remains relatively unexploited. Standalone inertial units are often created for riders and used in horse training. Nevertheless, IMU sensors can also be used as reliable sources for more tough challenges such as lameness determination (16). These phenotyping technologies are growing in importance due to their ability to generate real-time, non-invasive, and accurate animal-level information, enabling phenotyping on a large scale (17). Several IMU tools are available for equine gait analysis, each serving different purposes. One of the most used in research is the Equimoves (18) which measure gaits, detect lameness, and estimate speed by applying seven sensors, placed on the head, withers, sacrum and the four legs (19). Other IMU sensors focus specifically on detecting asymmetries in movement, which are therefore highly useful in clinical and veterinary applications. Nevertheless, most of the studies on the comparison between experienced clinicians and IMU evaluation suggested that IMU can strengthen but not replace subjective lameness assessment since the agreement was not always close to unity (20, 21). Finally, it has been shown the potential of IMUs for the evaluation of the horse–rider interaction during dressage riding, training of horses, or coaching (22). The overall advantage of implementing the use of IMU in horses is the possibility of gathering objective movement data in field conditions, where the use of well-established methods like OMC would be impractical and extremely expensive. In addition, since the technology is rather simple to implement, there is the possibility to collect objective data on a large scale of horses. However, IMUs also have some disadvantages, one of which is represented by their usually high cost, which has, nevertheless, decreased in recent years. In addition, to obtain reliable and reproducible data, care must be taken in placing the sensors in the right position, therefore, if not correctly implemented, this technology is also not free from human errors (22).

**Figure 1:**
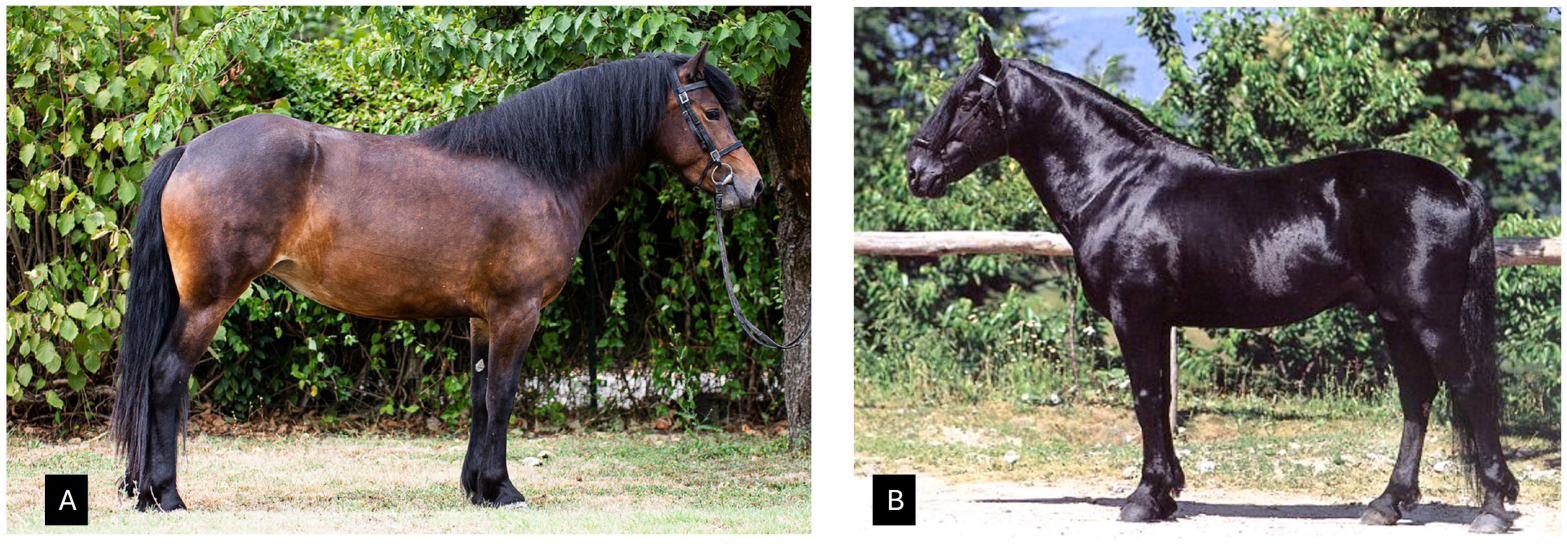
Example of Bardigiano **(A)** and Murgese **(B)** horse.

Therefore, in this article we focus on addressing the above-mentioned gap in knowledge by studying horses’ gaits via IMU sensor data. We aim to investigate on IMU recorded data (i) the influence of environmental factors and biometric measurements, (ii) their repeatability, (iii) the correlation with judge evaluations, and (iv) their predictive value. To achieve this, we focused on two Italian horse breeds: the Bardigiano and the Murgese. The Bardigiano, bred in North Italy, was used in the past for meat production and is considered a meso-brachymorphic horse (Figure 1A). The term meso-brachymorphic describes a body type that is medium-sized, with relatively short limbs and a robust, muscular structure. In horses, a meso-brachymorphic horse like the Bardigiano typically has a robust, muscular body with a wide chest, strong limbs, and a more compact appearance. The angles of the joints are very closed. This type of conformation is associated with strength and endurance rather than speed, making such horses well-suited for tasks like trekking, carrying loads, or agricultural work. The Murgese is bred in South Italy historically for agricultural work, its morphology is substantially different and considered as a meso-dolichomorphic horse (Figure 1B). The term meso-dolichomorphic describes a body type that is medium to tall with a long, lean frame. In horses, a meso-dolichomorphic type, typically has a leaner, taller structure with longer legs and a more refined body shape compared to meso-brachymorphic type (as the Bardigiano horse breed). This conformation is often associated with agility and speed, making these horses well-suited for equestrian sports such as dressage, where longer strides and fluid movements are beneficial. Both breeds are currently facing the conversion from their original purposes to a new breeding objective to match the market demand. Bardigiano is evolving to fit working equitation and trekking activities, while the Murgese is aiming to fit equestrian purposes, such as dressage performance. The two analyzed breeds symbolize Italy’s equestrian tradition, revealing adaptability, tight connection with their respective landscapes, and potential to find a place in the current market demands. Through our study, we aim to provide novel tools to enhance the promotion of these equine breeds, ensuring their role in the modern equestrian landscape.

## 2 Materials and methods

### 2.1 Sampling

A total of 135, including 101 Bardigiano and 34 Murgese, born between 2000 and 2019, were tested. Those horses were born between 2000 and 2019 which ensures a range of ages that reflects the current genetic diversity and management practices of the breeds. Among these, 72 young horses aged 3 or 4 years old were tested during a n a 70-day performance test. During the 70-day performance test, horses were subjected to a controlled environment that consisted in the same feeding and same trainer as well as a standardized training protocol that allowed theminimization of external variables affecting horse performance. The horses were evaluated three times:

1. First evaluation, where a committee comprising a veterinarian, and two judges evaluated the overall health status of the horse and took biometric measurements.
2. A second trial at 30 days involved a session of free jumping, and an under-saddle session with a standardized trail to reduce environmental influence. Only one rider was allowed to ride all the horses in the designed riding center, conducting a 10-min session comprising two gaits: walk and trot.
3. At the end of the performance test period (70 days), all the horses underwent a second trial repeating the free jumping and under-saddle (ridden) tests, now including canter, and a draft trial was added only for the Bardigiano horses. The same rider of the first test was involved in the second trial. A panel of at least three judges and riders were asked to evaluate the horses based on the criteria outlined in Table 1 for each trial.

**Table 1:** Criteria evaluated by judges and riders during the performance test.

Trait	Judges’ evaluation	Riders’ evaluation
Daily management in the stable		✓
Acceptance of harnessing, mounting, docility in approaching the rider		✓
Technique on jumping – front passage	✓	
Technique on jumping – back passage	✓	
Rideability, response to rider commands, and attitude towards work	✓	
Trot – rhythm, impulsion, amplitude, elasticity, and regularity	✓	
Canter – rhythm, impulsion, amplitude, elasticity, and regularity	✓	
Obedience, and trust towards the rider during the exercise	✓	
Attention to requests	✓	
Free jumping	✓	
Flatwork	✓	
Draft test	✓	
Elevation at a trot		✓
Frequency at trot		✓
Symmetry		✓
Elevation at canter		✓
Frequency at canter		✓
Recovery time		✓

**Figure 2:**
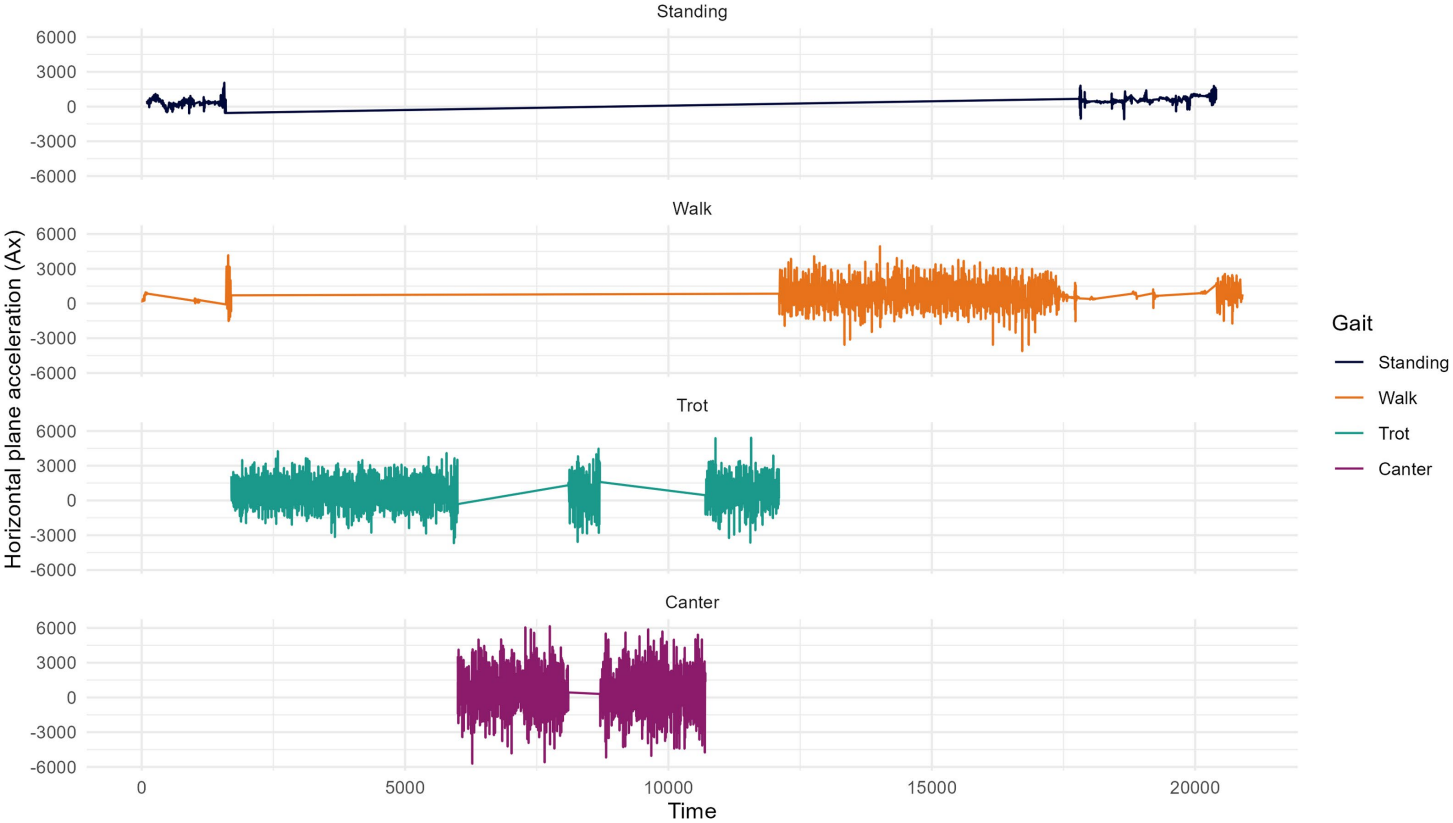
Example of data collected by x axis of the Equisense Motion S accelerometer. Data collected during the trial performed by the horses has been divided by gait.

The performance test was conducted over 3 years: 2020, 2021, and 2022 with testing periods in June, July, and August for the Bardigiano in two different riding centers and November, December, and January for the Murgese, in three different riding centers. Horses experiencing veterinary issues before or during the test period were excluded from the study. For the 63 horses included in the study which were not sampled during a performance test, the same protocol was used for a total of 10 min trial (Supplementary Figure S1). In addition, a survey to collect animal and environmental factors was developed (Supplementary Figure S2). This protocol included information on sex, birth date, rider’s skills, biometric measurements (e.g., height at withers, thoracic circumference, cannon bone circumference, shoulder length), management practices, rider details, arena conditions, and health traits (Supplementary Figure S1). All gait measurements were conducted by the same operator using Equisense Motion S^®^, [Micromegas, Headquarters: 231 Allée Faust d’Elhuyard 64,210 Bidart, France] a 9-axis inertial unit equipped with an accelerometer (3 axes), a gyrometer (3 axes), and a magnetometer (3 axes); placement is shown in Supplementary Figure S3. The inertial system acquires 100 measurements per second, enabling precise analysis of the horse’s locomotion (Figure 2). Furthermore, by adding an electrode, the sensor measures horses’ heart rate during the session. The parameters collected by the IMU sensor and electrode included stride frequency, regularity, and elevation for walk, trot, and canter, as well as symmetry and weak diagonal at trot, heart rate, speed, and distance. A specific definition for each trait is reported below:

- Stride frequency refers to the number of complete strides a horse takes per minute, where a stride is the sequence of hoof lifts and placements of the same limb;
- Regularity measures the consistency of gait rhythm and is scored on a scale from 0 to 10, where 10 indicates perfect consistency;
- Elevation is the vertical displacement of the horse’s body during each stride, measured in centimeters;
- Symmetry is evaluated while the horse trots in a straight line, comparing the lengths of paired strides. It is scored on a scale of 0 to 10;
- Heart rate: the rhythm, in beats per minute (BPM), that beats the heart of the horse.

Distance and speed were not considered in this study since they rely on data collected via GPS which is not included in the Equisense Motion S® but only provided as additional data if during the riding session the rider wears a phone.

### 2.2 Statistical analysis

#### 2.2.1 Analysis of variance

The effect of environmental factors and biometric measurements was assessed through an analysis of variance (ANOVA), performed using R (v.4.1.2), with the following model:

$\begin{array}{c} Y_{\mathrm{ijklmn}}=\mu +\mathrm{Bree}d_{i}+Sex_{j}+Age_{k}+\mathrm{Shoein}g_{l}+\mathrm{Ride}r_{m}\\ +\;\mathrm{Training}\;\mathrm{leve}l_{n}+\mathrm{Heigh}\;\mathrm{at}\;\mathrm{withers}\;\mathrm{within}\;\mathrm{bree}d_{i}\\ +\;\mathrm{Cannon}\;\mathrm{bone}\;\mathrm{within}\;\mathrm{bree}d_{i}\\ +\;\mathrm{Shoulder}\;\mathrm{lenght}\;\mathrm{within}\;\mathrm{bree}d_{i}+\varepsilon_{\mathrm{ijklmn}} \end{array}$

Where:

-
$Y_{\mathrm{ijklmno}}$ is the observed gait trait via the IMU sensor;
-
μ represents the intercept of the model;
-
$\mathrm{Bree}d_{i}$ is the effect of the breed (Murgese or Bardigiano);
-
$Sex_{j}$ represents the effect of the horse’s sex (male or female);
-
$Age_{k}$ represents the effect of the horse’s age (young ≤4; adult >5);
-
$\mathrm{Shoein}g_{l}$ is the effect of the shoeing (shod, forelimb shod or not shod);
-
$\mathrm{Ride}r_{m}$ indicates the effect of the rider’s level (beginner, intermediate or expert) based on the rider’s license;
-
$\mathrm{Training}\;\mathrm{leve}l_{n}$ is the effect of the horse’s training (defined in hours per week: 0–2, 3–4, >4);
- Height at wither within ${\mathrm{breed}}_{i}$ , Cannon bone within ${\mathrm{breed}}_{i}\text{,}$ Shoulder length within ${\mathrm{breed}}_{i}$ effect of the interaction between breed and biometric measurements (divided into quartile classes).
- ɛ_ijklmn_ is the error term.

A total of 134 horses were evaluated for this analysis. Post-hoc Tukey contrast tests were conducted to identify pairwise differences between group means using the base R Tukey HSD function.

#### 2.2.2 Repeatability

The repeatability of the horse’s performance was assessed using the rptR package in R (v.4.1.2), on a subset of data comprising 47 Bardigiano horses participating in the performance test. All horses involved in repeatability calculation were female, aged 3 or 4 years, untamed when the performance test started. The evaluation took place after 30 days and 70 days of training, with assessments conducted using both judges’ evaluations and Equisense Motion S® data. The repeatability was assessed only on the gaits shared by the two trials: walk and trot, using the following formula (23):


$$R=\frac{V_{G}}{V_{G}+V_{R}}$$

Where R is the repeatability, $V_{G}$ is the variance among group means while $V_{R}$ is the residual variance at data level.

#### 2.2.3 Correlation

A subset of 111 trials was selected to evaluate the correlation between judge evaluations and IMU sensor data, focusing on trials with both types of data available. This analysis was restricted to horses participating in the performance test due to the reliability of the judges’ and riders’ scores. Both the 30 and the 70-day trials were considered. Pearson’s correlation coefficient was used to assess the strength and direction of linear relationships between IMU sensor measurements and judges’ scores as well as within them.

#### 2.2.4 Predictive models

To determine the feasibility of predicting judge evaluations via Equisense Motion S^®^ objective traits, the judges’ evaluations were categorized into binary classes: ‘negative’ for scores below the mean and ‘positive’ for scores at or above the mean. The study employed the same dataset used for correlation analysis. Three different algorithms were employed to assess the prediction study: Support Vector Machine (SVM), Gradient Boosting Machine (GBM), and K-Nearest Neighbors (KNN). These algorithms were chosen due to their different classification approaches, aiming to identify the best fit for the data. The SVM works by finding a hyperplane that best separates data points belonging to different classes (24) GBM is a tree-based model where trees are built to correct errors from previous ones (25), and KNN is a non-parametric model classifying data points based on the majority of neighbor labels in the training data (26). The models were evaluated using a 10-fold cross-validation to increase the accuracy and reliability of the analysis. Only judge evaluations concerning behavior, walk, and trot traits were studied due to the completeness of the dataset. The three models were implemented in R (version 4.1.2), SVM via the e1070 package, KNN via the class package, and GBM via the caret package. Several metrics were considered to assess model performance (27), including accuracy (the ratio of correctly predicted instances to the total instances), sensitivity (the proportion of actual positive cases that were correctly identified), specificity (the proportion of actual negative cases that were correctly identified), and F1 score (the harmonic mean of sensitivity and specificity), calculated as follows:


$$F1=\frac{2\;x\;\mathrm{Precision}\;x\;\mathrm{Recall}}{\mathrm{Precision}+\mathrm{Recall}}$$

For the SVM model, the tune function in the e1070 package in R was utilized to tune the gamma, which represents the complexity of the decision boundary, and the cost parameters which represent the balance between margins and misclassification. In the GBM model, the number of trees, shrinkage value, interaction depth, and the minimum number of nodes were tuned for each trial. Specifically, the number of trees represents the total number of boosting stages, the shrinkage value controls the contribution of each tree, the interaction depth determines the maximum depth of the individual trees, and the minimum number of nodes specifies the minimum number of samples required to split a node. For the KNN model, the number of neighbors was tuned using the appropriate function in the class package. All parameters were tuned individually for each trial, and the details of the parameters used for tuning are provided in Table 2.

**Table 2:** Tuning parameters used for each trait and model.

Model	SVM		GBM				KNN
	Gamma	Cost	Number of trees	Interaction depth	Shrinkage	Min observations in node	*k*
Daily management	0.01	100	100	5	0.1	5	11
Acceptance of harnessing	0.000001	0.1	100	5	0.01	5	11
Rideability	1	1	100	1	0.1	5	11
Trot	0.000001	0.1	150	1	0.2	15	11
Obedience	0.001	100	150	1	0.01	10	11
Attention to requests	1	10	100	1	0.1	5	11
Flatwork	0.01	100	100	3	0.2	5	11
Elevation at a trot	0.1	1	50	1	0.2	5	11
Frequency at trot	0.01	100	50	1	0.2	5	11
Symmetry	0.01	100	50	3	0.2	15	11
Recovery time	0.1	100	50	3	0.2	5	11

## 3 Results and discussion

### 3.1 Analysis of variance

Table 3 presents the descriptive statistics for gait traits observed in horses participating in this study. Data on symmetry at trot and regularity were missing for some horses. This limitation likely arose from an insufficient training level for cantering and therefore a limited regularity or an inadequate duration of straight-line movement to capture symmetry data. Significant variability was observed in stride frequencies and elevations, reflecting both individual differences and breed-specific characteristics. As phenotypic variation results from the interaction between environment and genotype, the primary aim of this study was to identify environmental factors affecting gait traits (Supplementary Table S1).

**Table 3:** Descriptive statistics of gait traits collected by Equisense Motion S^®^.

Variable	*n*	Min	Max	Mean	SD
Frequency walk	135	39.33	57.31	51.66	3.32
Regularity walk	134	0.0	7.92	3.46	2.06
Frequency trot	134	78.32	109.33	90.27	6.04
Regularity trot	134	0.24	7.88	5.66	1.11
Frequency canter	132	79.63	112.15	99.73	9.24
Regularity canter	82	0.10	9.02	4.71	2.38
Elevation walk	135	1.13	6.46	2.85	0.84
Elevation trot	134	4.21	12.39	7.53	1.69
Elevation canter	132	7.40	22.33	15.92	2.54
Symmetry	96	1.75	8.70	6.88	1.17
Heart rate walk	108	34.4	121.92	93.28	14.21
Heart rate trot	104	89.84	157.69	126.86	13.14
Heart rate canter	100	108.73	196.67	156.83	18.95

**Figure 3:**
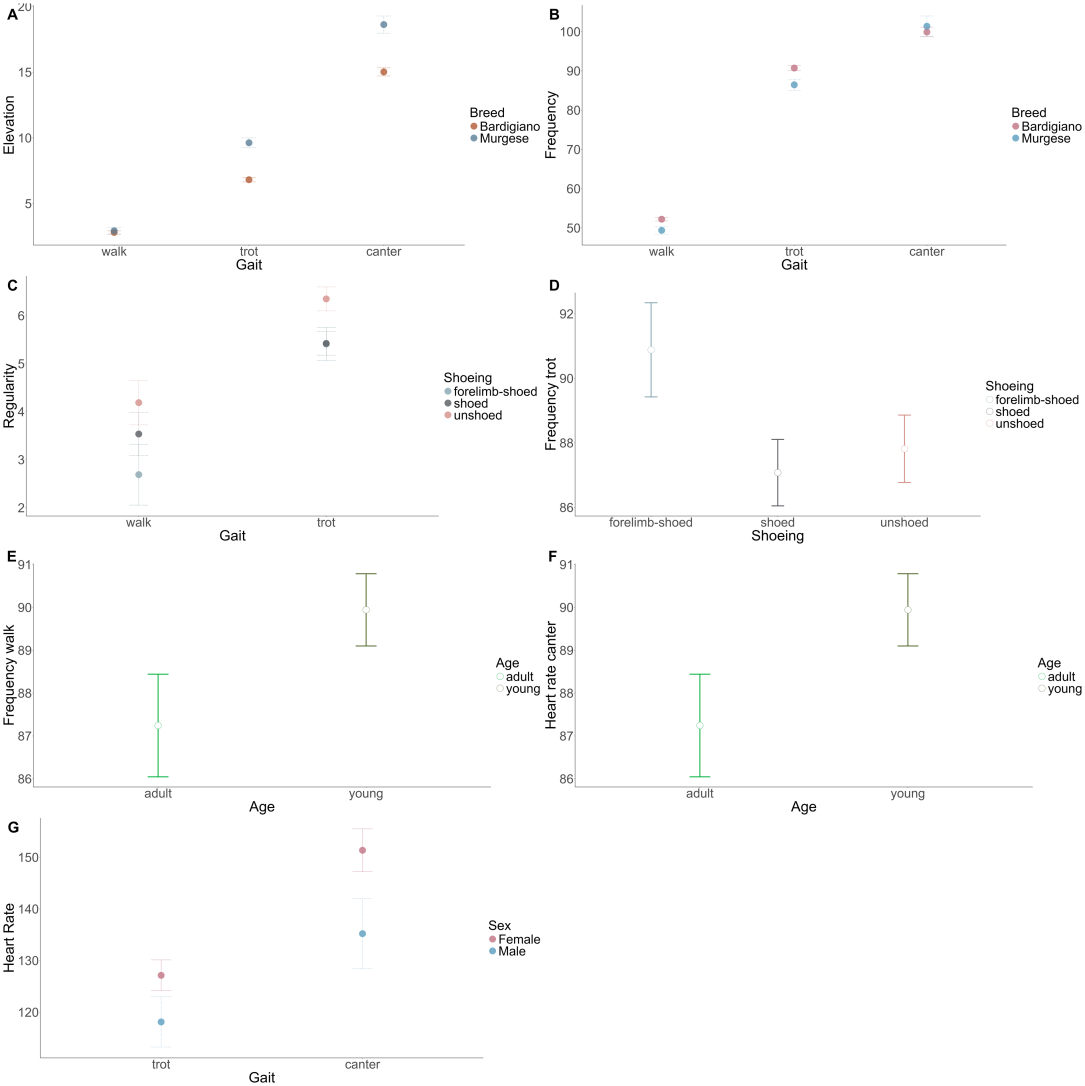
LSM (Least Squares Means) results show the effect of breed on elevation and frequency at the three gaits **(A,B)**, the effect of shoeing on regularity at trot and walk **(C)** and frequency at trot **(D)**, the effect of age on frequency at walk and heart rate at canter **(E,F)**, and the effect of sex on and heart rate at trot and canter **(G)**.

Among the factors investigated in the ANOVA, breed showed a significant influence on most of the gait traits measured by the sensor (*p* < 0.05) including elevation at trot and canter, stride frequency at walk, trot, and canter, as well as stride regularity at trot and canter. The significant effect of the breed highlights the different predispositions towards sporting activities and the distinct abilities between Bardigiano and Murgese. Indeed, the Murgese horses displayed on average greater elevation (+2.81 cm at trot, +3.60 cm at canter) (Figure 3A) and lower stride frequency (−2.83 stride/min at walk, −4.27 stride/min at trot) (Figure 3B). Based on those differences we can hypothesize the enhanced potential for sporting activities of Murgese horses, likely due to their physical attributes and selection towards dressage performance. As an example, a higher elevation is particularly valued in dressage competitions, thus, it is not surprising that it is higher in the Murgese breed. On the other hand, Bardigiano horses displayed lower elevation and higher frequency at walk and trot, which are traits favorable for endurance activities such as trekking or working equitation, where energy preservation is essential. The opposite trend was shown at canter, where the Bardigiano horses showed lower frequency (−1.46 stride/min). However, this observation may be influenced by the higher proportion of adult and well-trained Bardigiano horses (50% compared to 26% in Murgese) and should be interpreted with caution. Furthermore, it was observed that un-shod horses showed a greater stride regularity at walk (+1.50) and trot (+0.37) (Figure 3C), along with reduced gait frequency at trot (−3.07 stride/min) (Figure 3D) compared to forelimbs shod horses. This latter result suggested the potential benefits of natural balance and enhanced gait expression in un-shod horses especially compared to front limbs shoed horses. This finding aligns with existing knowledge that shoes can alter gaits, since joint angles of the pastern move differently between shod and un-shod horses (28) as well as that shoes’ mass can influence gait (29). Horses only shod in the forelimbs showed lower values of regularity (−0.85 compared with shod and − 1.5 compared with un-shod; *p*-value <0.0001) (Figure 3C), possibly due to the increased difficulty in balancing and maintaining stable gait during the session, as the center of gravity shifts unnaturally towards the hind legs. Despite shoes being applied to protect against the wear of the hoof wall, to improve performance and to provide additional support on slippery surfaces, they may restrict the hoof mechanism and add additional weight on the distal limb. This increases its inertia, demanding a higher energy expenditure in protracting and retracting the limbs. Thus, the weight of shoes is likely affecting gaits, altering both energy and kinematics of locomotion (30).

The age (Figures 3E,F) had a significant effect on the frequency of walk, by indicating that adult horses (−2.91 stride/min) are commonly better-trained and exhibit lower gait frequency during the session. Additionally, canter heart rate was impacted by age, with younger horses having higher heart rates (+32.02 bits/min), which suggests that training level affects parameters such as the cardiovascular response of horses. The sex had a significant effect on heart rates both at trot and canter (Figure 3G), with males showing a lower heart rate at trot (−9.03 bits/min) and canter (−16.10 bits/min) compared to female. This result may be attributed to pre-selection and increased attention given to training of male horses. Typically, in these breeds, only a few stallions undergo training under the saddle, leading to a pre-selection process to identify the most valuable ones.

Surprisingly, rider experience and horse training level, along with cannon bone circumference and shoulder length, did not yield significant effects on gait traits, likely due to the limited variability in these factors in our samples. Indeed, most of the horses were trained by only two professional riders following the same training routine. Regarding biometrical measurements, they did not provide any significant effects, probably because their variability is already included in the breeds’ variability.

### 3.2 Repeatability

The study’s second aim was to assess which traits change or stay consistent during the horse’s life. This information can provide a better understanding of the effect of training on traits improvements and the identification of traits bound to horse’s natural attitude. Stride regularity at walk (0.635) and elevation at walk (0.717) demonstrated the highest repeatability between the two trials, indicating that walk is less influenced by training and remains relatively consistent throughout the horse’s life. Conversely, all the other measurements had lower repeatability, suggesting greater susceptibility to environmental influences and the potential for improvement through training (Supplementary Table S2).

### 3.3 Correlation

**Figure 4:**
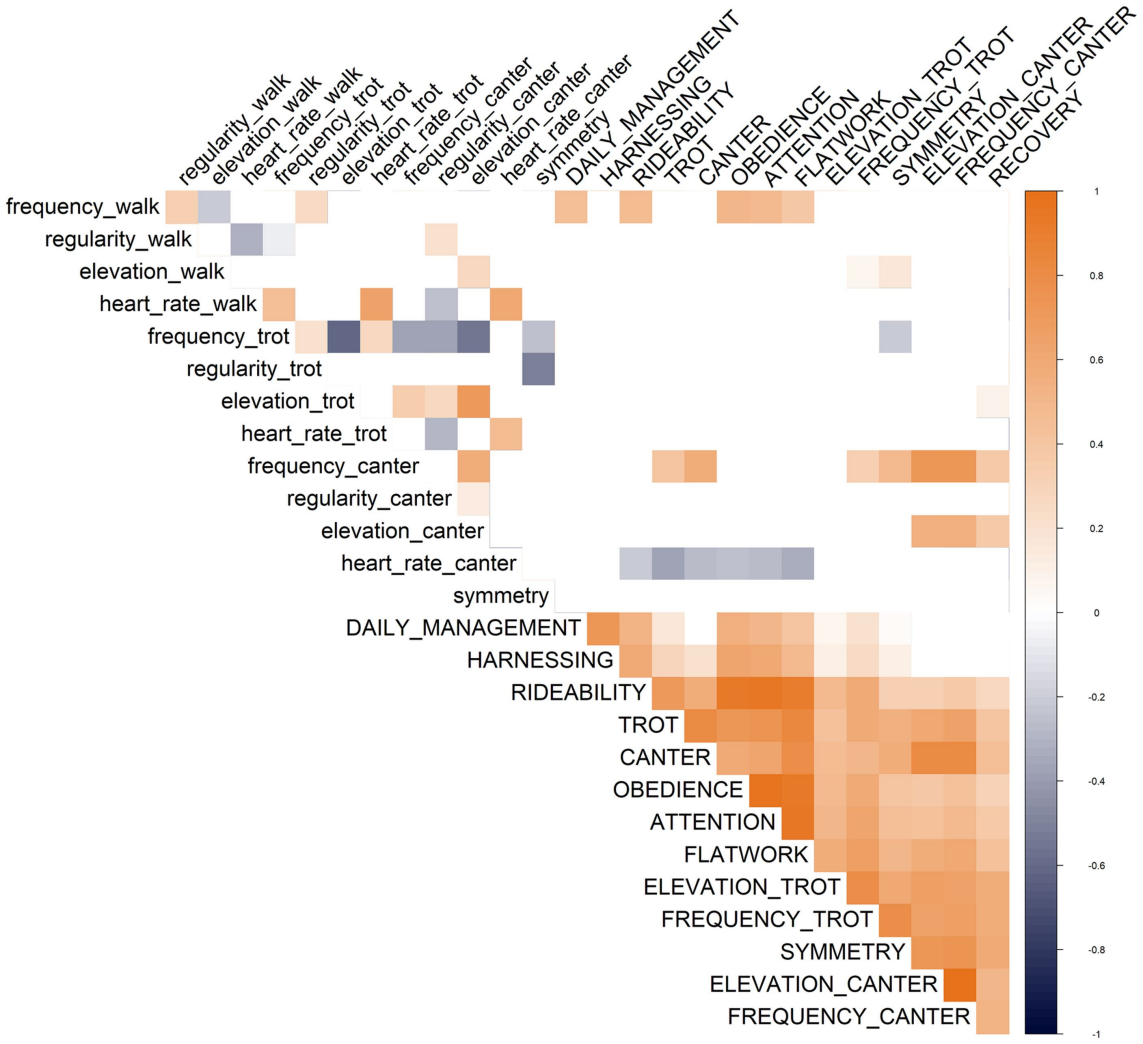
Correlation plot. Displays significative correlations between judges’ evaluations (uppercase) and sensor data (lowercase). Positive correlations are in orange, while negative correlations are in blue with transparency reflecting correlation’s strength.

Another key aspect when using IMU sensor data collection is to assess how those new traits correlate with traditional evaluation. Correlation analyses within sensor data and between judges’ evaluations and Equisense Motion S^®^ performance revealed interesting patterns. Hereafter and in Figure 4, only significant correlations are reported and further discussed. Within sensor data, elevations showed positive correlations among gaits, ranging from high for trot-canter (0.618) to moderate for walk-canter (0.250); this can be due to the horse’s training or the rider’s attitude to collect the gait. However, since the ANOVA did not highlight any significant difference between riders for the elevation, the differences might be bound to the natural predisposition of the horse. Further studies are needed to investigate this aspect; one potential solution is to study elevation without the rider to truly understand the cause of this correlation. Similarly, heart rate exhibited strong positive correlations among gaits, ranging from trot-canter (0.795) to walk-trot (0.720), indicating that horses’ fitness level affects heart rate across all gaits. Stride frequency at trot negatively correlated with elevation at trot (−0.448) and canter (−0.482), suggesting that horses with higher frequencies may expend energy on increasing frequency rather than increasing elevation which is a proxy of gait quality. This may be perceived from a rider’s perspective as the tendency to hurry the trot, which is usually considered a negative aspect since it does not create momentum and energy usable for sports activities like jumping or dressage. This trend may be influenced by breed traditional use as those historically used for draft work prioritize forward movement over vertical collection. Conversely, stride frequency at canter is positively correlated with elevation at trot and canter (0.426, 0.303), indicating that increasing stride frequency likely leads to increased elevation and overall gait activity and quality. In small breeds like the Bardigiano horse, the activity of the gait is considered positive and usually is described by the rider as a movement of the body weight on the back limbs, which can lead to an improved propulsion forward and upward.

The correlations between judge or rider scores and sensor data reveal interesting results for both general traits such as rideability and obedience, as well as specific ones like flatwork, trot, or elevation at trot and canter. Rideability correlates positively with stride frequency at walk (0.255) indicating that judges perceive a better work attitude in horses with good activity at walk. A negative correlation was found between rideability and heart rate at canter (−0.318); this suggests that horses with lower heart rates during canter tend to be more rideable and easier for the rider to manage. Similarly, obedience and trust towards the rider during exercises moderately correlate with stride frequency at walking (0.275) and negatively with heart rate at canter (−0.304), suggesting an overlap between the evaluation of rideability and obedience. Elevation at canter, measured by the sensor, exhibited positive correlations (from 0.374 to 0.471) with the evaluation provided by the rider regarding impulsion at canter, frequence at canter and effort recovery time. This suggests riders’ capability to discriminate overall gait quality, providing a positive score for horses that are engaging the back limbs in several canter related evaluation. The positive correlation of elevation at canter expressed by the sensor with recovery time expressed by the judges (0.374) can be explained through the association between higher stride and increased energy expenditure with a consequent increase in heart rate, resulting in a longer recovery time. Conversely, the correlation between the elevation measured by the sensor and the evaluation provided by the judges regarding elevation (0.461) and frequency (0.471) indicates a lack of differentiation by riders’ scores between the two measurements. Judges tended to unify the two results, considering the overall quality of the canter gait and giving a positive score in both elevation and frequency if the horse reveals a high elevation at canter, recorded by the sensor. Regarding elevation at trot from sensor data, it only correlates significantly with recovery time (0.263), suggesting that higher elevation requires more effort for the horse, resulting in a longer time needed to recover the energy perceived by the rider. Heart rate during canter displayed negative correlations (ranging between −0.284 to −0.352) with the evaluation of rideability, trot, canter, obedience, and flatwork. This suggests that an increased heart rate during canter results in reduced rideability, obedience, and overall gait quality assessed by judges’ scores. Conversely, stride frequency at walk showed moderate positive correlation (ranging from 0.235 to 0.280) with management of the horse, rideability, obedience, and flatwork, reflecting the perception of an active, obedient, and responsive horse engaging its hindlimbs and ready to respond to the riders’ requests.

Regarding the correlation within judges’ and riders’ evaluation, a highly positive correlation between obedience and rideability (0.914) implies that these evaluations may measure the same aspect of the horse’s behavior. Therefore, it may be helpful to unify these scores into a single evaluation assessing the overall attitude of the horse towards collaboration with the rider, simplifying the assessment process for judges and ensuring consistency in evaluations. The strong positive correlation between elevation and frequency at canter (0.983) provided by the rider indicates riders’ difficulty in objectively discriminating those two traits. Riders often evaluate positively a horse exhibiting an active gait, characterized by both good elevation and frequency. This preference aligns with the improvement that is sought for breeding purposes, such as for the Bardigiano horse, that originally was bred for agricultural purposes; thus, an active canter is not common and at the same time appreciated by the judges.

### 3.4 Predictive models

**Figure 5:**
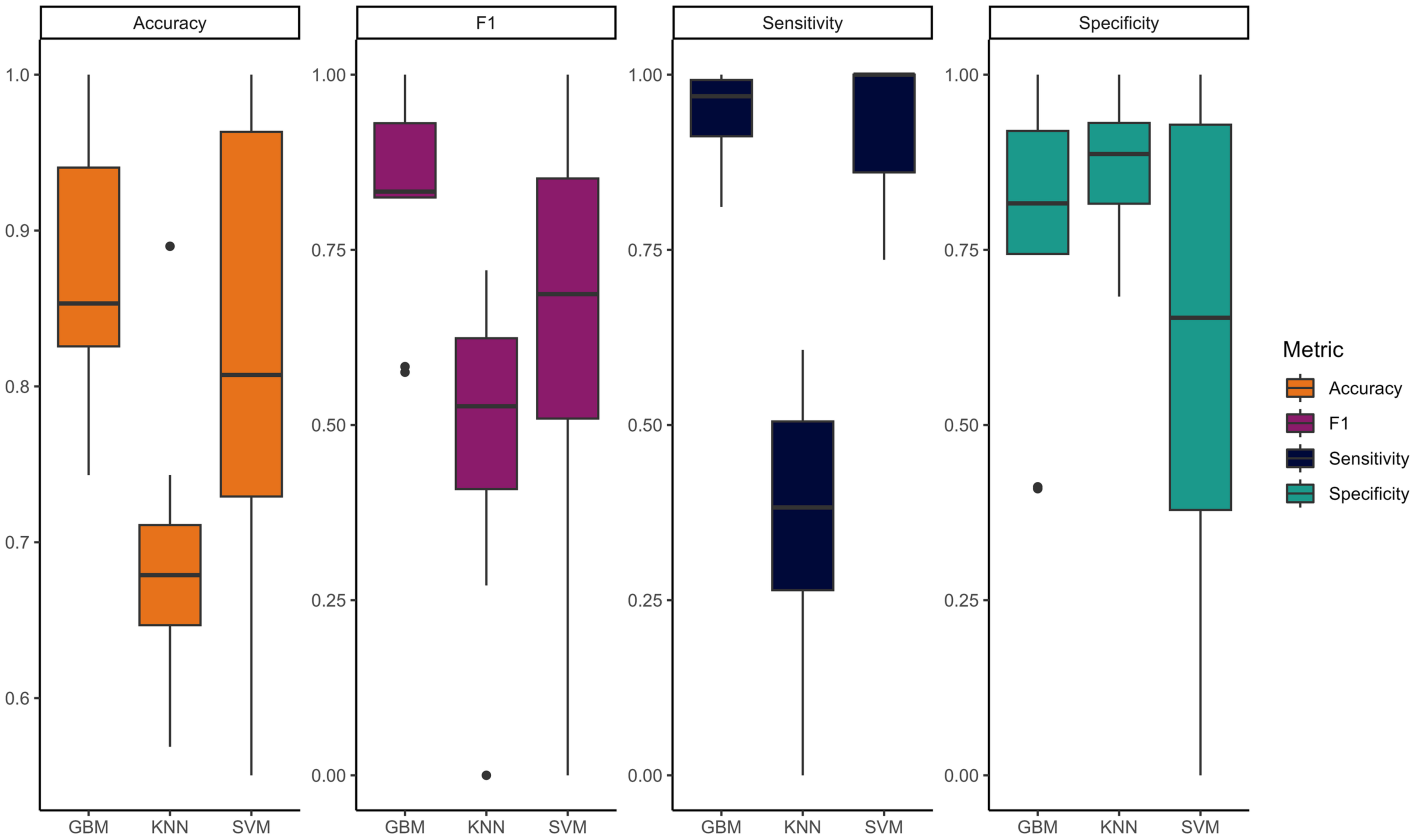
Overall model performance comparison through different metrics.

To explore the predictive potential of sensor data for evaluation outcomes, three different models were tuned, used and their performance measured within a 10-fold cross-validation. Among the three models tested, the GBM model achieved the highest accuracy and lowest error, with its F1 score consistently surpassing those of the other models (Figure 5).

The SVM model has consistently shown the widest range of all classification performance metrics. Accuracy for the SVM model ranged from 55 to 100%, while GBM and KNN models demonstrated higher consistency, with accuracies from 74 to 100% for GBM and 64 to 88% for KNN (Supplementary Table S3). Despite reaching higher accuracy for some traits, the F1 scores of the SVM models were generally lower, due to a lower specificity. This suggests a tendency to classify all cases as positive; this possibly is due to its susceptibility to unbalanced classes (31), also indicating that our classes may lack clear separation and seem to overlap. Indeed, this model is better suited for classification tasks with distinct class boundaries. The KNN model showed consistency and tended to achieve a high level of specificity but had the lowest sensitivity, which indicates difficulty in detecting positive cases while correctly identifying negative ones. These results also suggest sensitivity to irrelevant features, highlighting the need for careful feature selection and training samples to improve performance, as already proved by several studies (26, 32, 33). In our data this is highlighted by the predictive trials that exhibited lowest sensitivity scores, such as those assessing daily management, rideability, obedience and attention to requests. These traits lack clear sensor-collected values and are objectively more challenging to detect solely through sensor data.

The evaluations predicted with the highest overall accuracy were rideability, attention to the requests, and recovery time, with respective accuracies of 83% (F1 = 0.74), 85% (F1 = 0.75), and 90% (F1 = 0.90). This indicates that we can correctly predict over 80% of the judges’ scores using IMU measurements. The closeness between the accuracy in % and the F1 Score suggests that the misclassified results will be equally distributed between False Positive and False Negative, leading to a balanced model.

Focusing on the best model (GBM), the highest accuracies (100%) were observed for daily management, flatwork, and recovery time, followed by an accuracy of 88% for attention to requests and trot trial. Although 100% accuracy must be interpreted with caution, as it may indicate overfitting, it could still be a realistic prediction for very small datasets and easily predictable trials. Indeed, a precise collection of heart rate data makes recovery time easy to predict, similarly the flatwork score should be straightforward to predict since the evaluation is based on frequency, elevation, and regularity. All F1 scores were above 0.85. Some results such as those for trot or flatwork and recovery time were expected due to the direct link of IMU data and judges’ evaluations since they are evaluating the same aspects. Oppositely, the impact of daily management and attention to requests on the traits measured by the sensor is not straightforward and may lead to poor classification results. However, it appears that gait parameters somehow predict aspects related to the horse’s behavior and collaboration with humans. A possible explanation would be that the rider’s corrective actions after an unexecuted request may influence the overall balance of the horse and its natural gait, interfering with the sensor-measured traits.

Although these results are preliminary and judges’ evaluations were divided into only positive or negative scores, there seems to be the possibility to predict judges’ scores from sensor data. With more data collected in the future, it might be possible to predict judges’ scores through portable and easy to use sensor data, potentially reducing human error and providing owners or buyers with a more precise way to evaluate animals. This cost-effective method could allow for the evaluation of more animals, aiding in selecting horses that better meet the desires of future owners regarding behavior, dressage performance, or recovery capability, aspects which are increasingly important.

## 4 Conclusion

In conclusion, this research aimed to study gait traits using sensor data collected via Equisense Motion S^®^. Differences in several gait traits were identified, highlighting the different predispositions between the Bardigiano and Murgese. These differences emphasize the importance of preserving local breeds, as they possess unique gait traits that are essential for maintaining their genetic and functional diversity. Factors such as shoeing and age showed an effect on most of the gait traits collected via Equisense Motion S®. In contrast, riders’ skills and horses’ training levels did not significantly influence gait traits, possibly due to the homogeneity of our samples. It was also observed that only the walk gait trait remained consistent across the two trials, suggesting that other gait traits may be more susceptible to variations due to training. Most of the judges’ evaluations are correlated to sensor data although some of them were strongly related to each other. This suggests that judges often assess overall gait quality rather than focusing on specific traits. This reinforces the value of sensor data for detailed analysis of gait traits, which are challenging to assess accurately with the human vision alone. Sensor data allowed for accurate prediction of judges’ evaluations, demonstrating the potential of this technology for reliable performance assessment. In conclusion, the integration of sensor technology provides valuable insights into horse’s performance evaluation, with implications for breeding, training, and competitive sports. This technological advancement can help the breeders in identifying horses with higher rideability potential, thereby accelerating the selection process.

## Data Availability

The original contributions presented in the study are included in the article/Supplementary material, further inquiries can be directed to the corresponding author.
