# Supplementary material for: Inertial measurement unit technology for gait detection: a comprehensive evaluation of gait traits in two Italian horse breeds
Source: Front Vet Sci. 2024 Oct 16;11:1459553. doi: 10.3389/fvets.2024.1459553 (PMC11521968; doi:10.3389/fvets.2024.1459553)
Supplement: Supplementary file 2 [file Data_Sheet_2.docx]

Supplementary Material

# Supplementary Figures

| **MOVEMENTS** | | |
| --- | --- | --- |
| 1 | A | Enter in trot |
|  | X | Halt - Immobility - Salute |
|  | C | Proceed in trot - Track to the right |
| 2 | from M to M | Proceed in trot |
| 3 | M - X- K | Diagonal (trot) |
| 4 | From K to K | Proceed in trot |
| 5 | Between A and F | Transition at left canter - two laps |
| 6 | M | Transition at trot |
| 7 | M-C-H | Trot |
| 8 | H-X-F | Diagonal (trot) |
| 9 | Between A and K | Transition at right canter - two laps |
| 10 | H | Transition at trot |
| 11 | H-C-M | Trot |
| 12 | M-X-K | Diagonal (trot) |
| 13 | A | Walk - two laps |
| 14 | A | Down the center line |
|  | X | Halt - Immobility - Salute |

Supplementary figure 1: Trial performed during the performance test


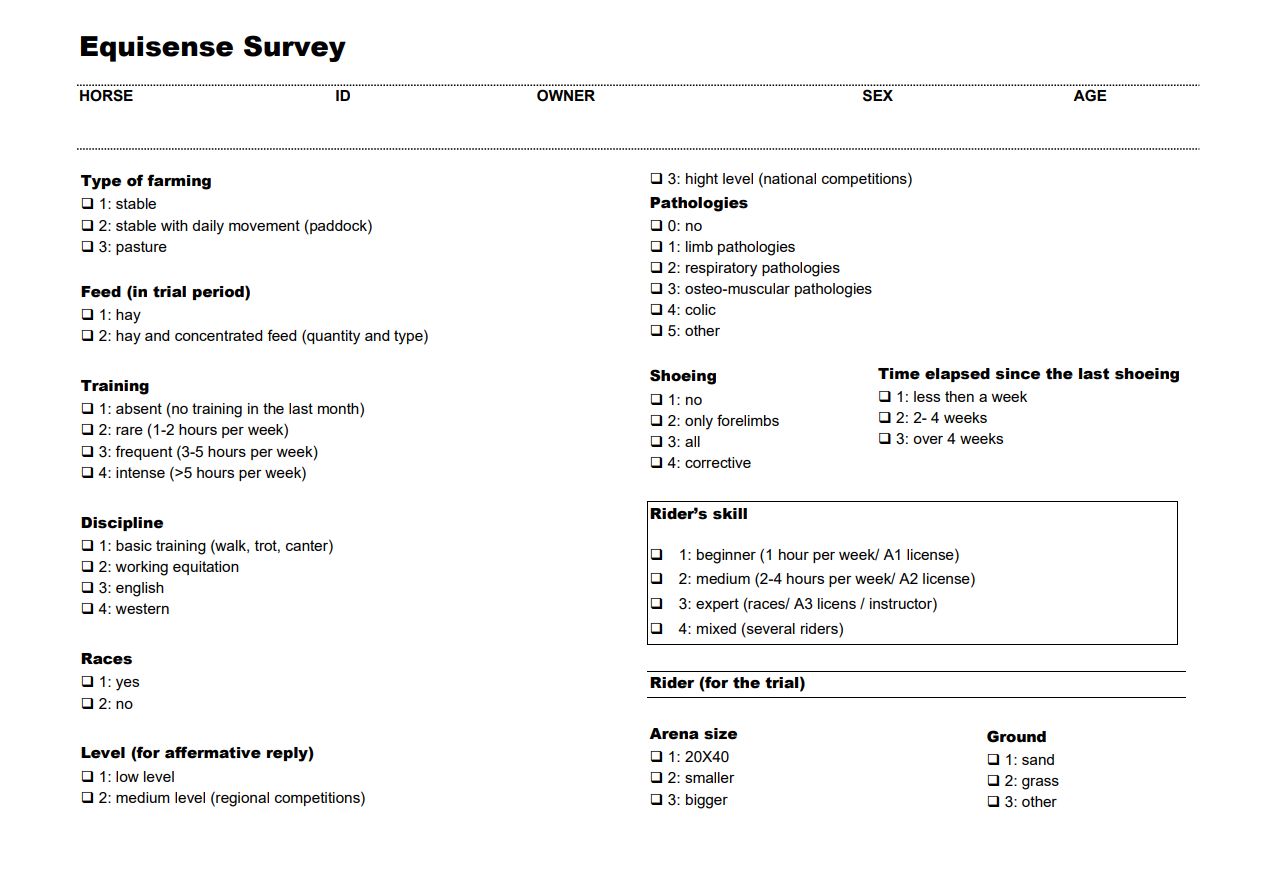


Supplementary figure 2: Survey used to evaluate animal and environmental factors


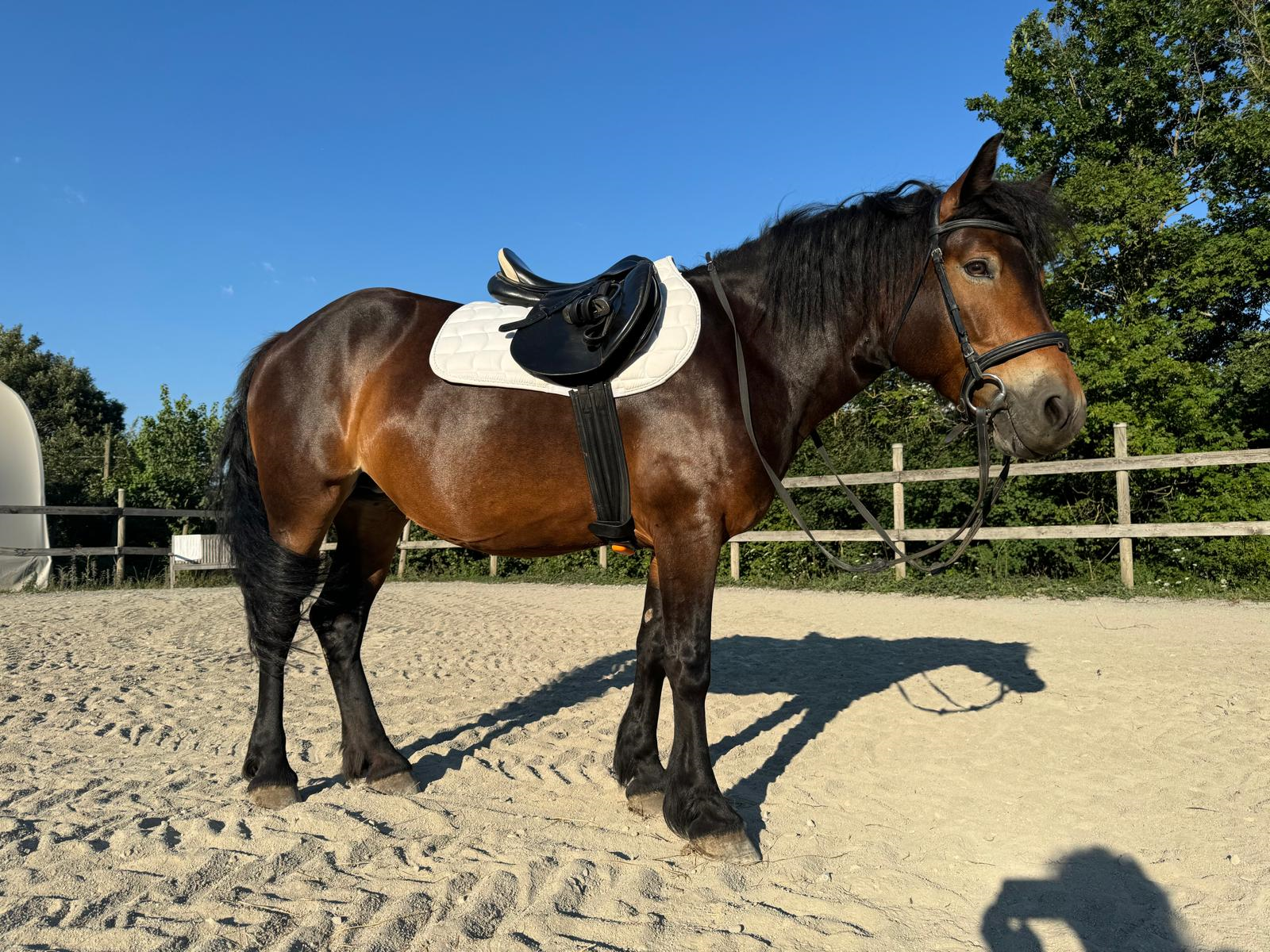

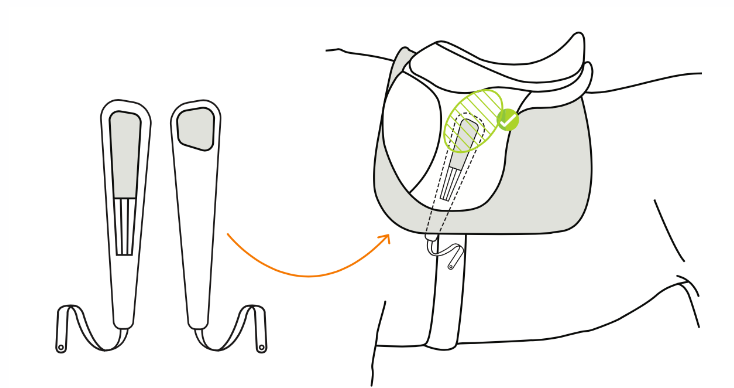


A

B

Supplementary figure 3: Sensor placement: A: attachment position, this part has to be fastened to the girth. B: electrode position, placed vertically under the saddle blanket.

# Supplementary Tables

Supplementary table 1: ANOVA results. Table 4: ANOVA results assessing the influence of various traits on horse gaits. F value is shown as well as the significance, coded as follow: ''***” (p ≤ 0.001), ''**” ( 0.001 < p ≤ 0.01), ''*” (0.01 < p ≤ 0.05), and ''. '' (0.05 < p≤0.1).

|  | Breed | Sex | Age | Shoeing | Rider | Training level | Height within breed | Cannon bone within breed | Shoulder length within breed |
| --- | --- | --- | --- | --- | --- | --- | --- | --- | --- |
| Frequency walk | 10.13** |  | 6.99** |  |  |  |  |  |  |
| Regularity walk |  | 4.51** |  | 7.46*** |  |  |  |  |  |
| Frequency trot | 48.65*** |  |  | 23.02*** |  |  |  |  |  |
| Regularity trot |  |  |  | 5.20** |  |  |  |  |  |
| Fequency canter | 4.32* |  |  | 13.18*** |  |  |  |  |  |
| Regularity canter | 8.80** |  |  |  |  |  |  |  |  |
| Elevation walk | 2.86. |  |  |  |  |  |  |  |  |
| Elevation trot | 118.46*** |  |  | 5.09** |  |  |  |  |  |
| Elevation canter | 56.66*** |  |  |  |  |  |  |  |  |
| Symmetry |  |  |  |  |  |  |  |  |  |
| Heart rate walk |  |  |  | 4.41* |  |  | 2.85* |  |  |
| Heart rate trot |  | 4.82* |  | 5.52* |  |  |  |  |  |
| Heart rate canter |  | 6.91 * | 5.17* | 9.31*** |  |  | 2.47. |  |  |

Supplementary table 2: Repetability IMU data between trial 1 (30-days) and trial 2 (70-days).

| trait | repetability | se |
| --- | --- | --- |
| stride_frequency_walk | 0.37 | 0.153 |
| stride_regularity_walk | 0.635 | 0.115 |
| stride_frequency_trot | 0 | 0.104 |
| stride_regularity_trot | 0.106 | 0.136 |
| elevation_walk | 0.717 | 0.089 |
| elevation_trot | 0.133 | 0.142 |
| symmetry |  |  |
| heart_rate_walk | 0.066 | 0.123 |
| heart_rate_trot | 0.053 | 0.129 |

Supplementary table 3: Metrics achieved for each model across the evaluated traits.

|  | GBM | | | | KNN | | | | SVM | | | |
| --- | --- | --- | --- | --- | --- | --- | --- | --- | --- | --- | --- | --- |
| Trait | Acc | Sens | Spec | F1 | Acc | Sens | Spec | F1 | Acc | Sens | Spec | F1 |
| Daily management | 1 | 1 | 1 | 1 | 0.89 | 0 | 1 | 0 | 0.94 | 1 | 0.42 | 0.59 |
| Acceptance of harnessing | 0.82 | 1 | 0.41 | 0.58 | 0.72 | 0.38 | 0.88 | 0.53 | 0.69 | 1 | 0 | 0 |
| Rideability | 0.85 | 0.92 | 0.74 | 0.82 | 0.64 | 0.26 | 0.89 | 0.4 | 1 | 1 | 1 | 1 |
| Trot | 0.88 | 0.93 | 0.82 | 0.87 | 0.57 | 0.43 | 0.68 | 0.53 | 0.55 | 1 | 0 | 0 |
| Obedience | 0.74 | 0.97 | 0.41 | 0.58 | 0.65 | 0.27 | 0.91 | 0.42 | 0.64 | 0.85 | 0.34 | 0.43 |
| Attention to requests | 0.89 | 0.98 | 0.74 | 0.85 | 0.69 | 0.28 | 0.95 | 0.43 | 1 | 1 | 1 | 1 |
| Flatwork | 1 | 1 | 1 | 1 | 0.68 | 0.16 | 0.96 | 0.27 | 0.83 | 0.97 | 0.58 | 0.71 |
| Elevation at a trot | 0.83 | 0.81 | 0.84 | 0.83 | 0.66 | 0.55 | 0.77 | 0.65 | 0.81 | 0.74 | 0.88 | 0.69 |
| Frequency at trot | 0.84 | 0.9 | 0.78 | 0.83 | 0.7 | 0.51 | 0.85 | 0.64 | 0.77 | 0.87 | 0.65 | 0.68 |
| Symmetry | 0.83 | 0.82 | 0.83 | 0.83 | 0.64 | 0.5 | 0.78 | 0.61 | 0.78 | 0.85 | 0.7 | 0.71 |
| Recovery time | 0.99 | 0.98 | 1 | 0.99 | 0.74 | 0.61 | 0.89 | 0.72 | 0.99 | 1 | 0.98 | 0.99 |
| Overall | 0.88 | 0.94 | 0.78 | 0.83 | 0.69 | 0.36 | 0.87 | 0.51 | 0.82 | 0.93 | 0.6 | 0.62 |
